# Primary head and neck cancer cell cultures are susceptible to proliferation of Epstein-Barr virus infected lymphocytes

**DOI:** 10.1186/s12885-022-10481-y

**Published:** 2023-01-13

**Authors:** Senyao Shao, Lars Uwe Scholtz, Sarah Gendreizig, Laura Martínez-Ruiz, Javier Florido, Germaine Escames, Matthias Schürmann, Carsten Hain, Leonie Hose, Almut Mentz, Pascal Schmidt, Menghang Wang, Peter Goon, Michael Wehmeier, Frank Brasch, Jörn Kalinowski, Felix Oppel, Holger Sudhoff

**Affiliations:** 1 Department of Otolaryngology, Head and Neck Surgery, Campus Klinikum Bielefeld Mitte, University Hospital OWL of Bielefeld University, Klinikum Bielefeld, Teutoburger Str. 50, 33604 Bielefeld, Germany; 2 Biomedical Research Center, Health Sciences Technology Park, University of Granada, 18016 Granada, Spain; 3 Department of Physiology, Faculty of Medicine, University of Granada, 18016 Granada, Spain; 4 CIBERFES, Ibs. Granada, San Cecilio University Hospital, 18016 Granada, Spain; 5 Center for Biotechnology (CeBiTec), Universität Bielefeld, Bielefeld, Germany; 6 Department of Pathology, Klinikum Bielefeld, Teutoburger Str. 50, 33604 Bielefeld, Germany; 7 Department of Otolaryngology Head and Neck Surgery, Peking University International Hospital, Peking University, Beijing, 102206 China; 8 Department of Laboratory Medicine, Klinikum Bielefeld, Teutoburger Str. 50, 33604 Bielefeld, Germany

**Keywords:** Head and neck cancer, Lymphocyte, Epstein-barr virus, Human papilloma virus, Primary cell culture model

## Abstract

**Background:**

New concepts for a more effective anti-cancer therapy are urgently needed. Experimental flaws represent a major counter player of this development and lead to inaccurate and unreproducible data as well as unsuccessful translation of research approaches into clinics. In a previous study we have created epithelial cell cultures from head and neck squamous cell carcinoma (HNSCC) tissue.

**Methods:**

We characterize primary cell populations isolated from human papillomavirus positive HNSCC tissue for their marker expression by RT-qPCR, flow cytometry, and immunofluorescence staining. Their sensitivity to MDM2-inhibition was measured using cell viability assays.

**Results:**

Primary HNSCC cell cultures showed the delayed formation of spheroids at higher passages. These spheroids mimicked the morphology and growth characteristics of other established HNSCC spheroid models. However, expression of epithelial and mesenchymal markers could not be detected in these cells despite the presence of the HNSCC stem cell marker aldehyde dehydrogenase 1 family member A1. Instead, strong expression of B- and T-lymphocytes markers was observed. Flow cytometry analysis revealed a heterogeneous mixture of CD3 + /CD25 + T-lymphocytes and CD19 + B-lymphocytes at a ratio of 4:1 at passage 5 and transformed lymphocytes at late passages (≥ passage 12) with CD45 + CD19 + CD20 + , of which around 10 to 20% were CD3 + CD25 + CD56 + . Interestingly, the whole population was FOXP3-positive indicative of regulatory B-cells (B_regs_). Expression of transcripts specific for the Epstein-Barr-virus (EBV) was detected to increase in these spheroid cells along late passages, and this population was vulnerable to MDM2 inhibition. HPV + HNSCC cells but not EBV + lymphocytes were detected to engraft into immunodeficient mice.

**Conclusions:**

In this study we present a primary cell culture of EBV-infected tumor-infiltrating B-lymphocytes, which could be used to study the role of these cells in tumor biology in future research projects. Moreover, by describing the detailed characteristics of these cells, we aim to caution other researchers in the HNSCC field to test for EBV-infected lymphocyte contaminations in primary cell cultures ahead of further experiments. Especially researchers who are interested in TIL-based adopted immunotherapy should exclude these cells in their primary tumor models, e.g. by MDM2-inhibitor treatment. BI-12-derived xenograft tumors represent a suitable model for in vivo targeting studies.

**Supplementary Information:**

The online version contains supplementary material available at 10.1186/s12885-022-10481-y.

## Introduction

Despite recent progress in cancer treatment, e.g. due to the development of immunotherapy and personalized medicine, the prognosis for many cancer patients remains devastating [1]. Understanding tumor biology is crucial to develop novel therapeutic strategies, prolong patients’ survival, and improve life quality. For basic and translational cancer research, in vitro and in vivo tumor models are needed which accurately reflect tumor biology. Primary tumor models directly established from surgically resected tissue represent a preferred approach, as research data indicates that these cells might reflect the original tumor’s properties much closer than decade old cell lines [2, 3]. However, primary cell culture implicates the risk of contamination with benign cell types. If unidentified, this may lead to experimental inaccuracies compromising the validity and reproducibility of research data [4–6].

Head and neck squamous cell carcinoma (HNSCC) originates from the mucosal epithelial cells of the oral cavity, pharynx, or larynx and is a life-threatening disease with a mortality of about 50% [7, 8]. Risk factors for HNSCC are alcohol and tobacco consumption, and a subset of cases is caused by the infection with high-risk human papilloma virus (HPV). Standard therapy includes surgical resection, radiotherapy, chemotherapy, and immunotherapy. Due to differences in patient-specific tumor characteristics, these treatments show varying efficiency and new therapeutic concepts to improve the patients’ outcomes are urgently needed. Thus, the so-called personalized therapy including molecular targeting is drawing more and more attention of clinicians and cancer researchers [9–11]. As a fundamental procedure of obtaining basic characterization of tumorigenesis and testing the efficiency of designed personalized treatment, primary tumor cell cultures provide preclinical models mimicking the patient-specific genetic and molecular features [12, 13]. For example, HNSCC spheroids were employed as model system in previous studies [6, 14, 15]. In general, tumor spheroids appear to comprise more undifferentiated cell populations than adherent cell cultures, which designates them as highly valuable in vitro system to conduct basic and translational cancer research [16].

It is known however, that distinct cell types contained in a solid tumor, both malignant cells and benign cells, can be present in primary tumor model systems in vitro and in vivo [4–6]. This can lead to experimental flaws compromising the validity and reproducibility of research data. Hence, we detected possible markers to test HNSCC cell cultures for contamination with stromal fibroblast-like cells and successfully established a primary HNSCC spheroid cell culture system, as described previously [6]. Here, we describe that within this culture system delayed proliferation of Epstein-Barr-virus (EBV) transformed lymphocytes represents another obstacle in spheroid culture using patient-derived HNSCC tissue and show that in HNSCC primary cell culture a deeper testing for lymphocyte markers and EBV is advised before using rapidly growing spheroids for research. Moreover, we emphasize the importance of testing for the EBV status before applying TILs in immunotherapy and its potential contribution on personalized cancer treatment.

## Methods

### Primary tumor cell culture

Primary cells of BI-12 were derived from a 49-year-old male diagnosed with an HPV-positive metastatic tonsil squamous cell carcinoma. The cells were cultured PneumaCult™-Ex Plus medium (PNEU medium, Stemcell Technologies, Vancouver, Canada) supplemented as described previously [6]. Adherent cells were detached using Accutase (Capricorn Scientific, Ebsdorfergrund, Germany). All experiments were conducted according to the declaration of Helsinki, and as approved by the ethics committee of the Ruhr-University Bochum (AZ 2018–397-1), as described previously [6].

### Drug treatment and cell viability assay

The MDM2 inhibitor HDM201 (all Selleck Chemicals, Houston, TX, USA) was applied at designed concentrations in growth medium, and non-treated control (NTC) was treated with solvent DMSO. 0.33 mg/mL 3-(4,5-Dimethylthiazol-2-yl)-5-(3-carboxymethoxyphenyl)-2-(4-sulfophenyl)-2H-tetrazolium inner salt (MTS) solution in PBS with 34.6 μg/mL phenazine methyl sulfate (PMS) was used to determine cell viability as described previously [17]. MTS stock solution was prepared with MTS (Promega, Madison, WI, United States) and PMS(Sigma-Aldrich) up to 2 mg/mL and 0.21 mg/mL, respectively. A Tecan Infinite 200 microplate reader (Männedorf, Switzerland) was employed to measure the absorbance at 490 nm, and that at 690 nm was measured simultaneously as debris reference, growth medium was used as blank control.

### Real-time quantitative PCR

Real-time quantitative PCR (RT-qPCR) analysis was performed as described previously [6] using whole RNA isolated from at least 2 × 10^5^ cells. Specific primers were used to analyze the expression of the epithelial markers Epithelial cell adhesion molecule (EpCAM) and cytokeratin-19 (CK19), stromal fibroblast marker THY1, stem cell marker ALDH1A1, B lymphocyte markers CD19 and CD20, T cell markers CD3G, CD4, CD8, CD25, and forkhead box P3 (FOXP3), adhesion cell marker claudin 1 (CLDN1), Epstein-Barr virus markers toward the *BamHI-W* region and the *EBNA-1* region, as well as the HPV *E7* fragment. p53 pathway-associated markers MDM2, p53, CDKN1A, NOXA, Bcl-2 were also tested for valuation of HDM201 treatment effect. All the primers mentioned above were listed in Suppl. Table S1.

### Flow cytometry

Fresh single cells were harvested from passage 5, 12, 20 and resuspended in PBS. Cells were subsequently stained with mouse antibodies anti-CD3-PC5.5, anti-CD3-FITC, anti-CD19-ECD, anti-CD20-APC, anti-CD25-PC5.5, anti-CD45-PC7, anti-CD56-PE, (Beckman Coulter, Brea, CA, United States) according to the manufacturer’s standard procedures. Measurement was performed using a Beckman Coulter Navios flow cytometer.

### Data analysis

The data was collected and summarized in Microsoft Excel, then presented to GraphPad Prism 8 software for subsequent plotting and testing. The half maximal inhibitory concentration (IC_50_) was calculated by the function of ‘log(inhibitor) vs. normalized response – Variable slope’, as well as the Hill Slope and R square for quality of curve fitting. The differences among groups were tested by functions of ‘one-way ANOVA’ or ‘non-parameter t test’. The quantified data were presented as ‘*mean* ± *SD.* The significant difference was defined as *p* < 0.05.

### Histology and Immunohistology

The primary tumor was fixed using 10% formalin and embedded in paraffin by an overnight routine embedding program of the Tissue-Tek VIP (Sakura, Umkirch, Germany). After the embedding process, the tumor is poured in molten paraffin to create a paraffin block to make sections. The tissue was sectioned by a sliding HM430 microtome (Zeiss) to receive 2 µm sections. HE staining was performed according to standard protocols using a linear COT 20 tissue stainer (MEDITE, Burgdorf, Germany).

Immunohistochemistry for p16 was performed according to the antibody protocol of the automated Omnis immunohistology system (Agilent DAKO, Santa Clara, CA, United States) using an mouse-anti-p16 primary antibody, JC2, 1:50 (ZETA Corporation, Agilent), an HRP-conjugated secondary antibody, and DAB chromogen. Indirect immunofluorescence staining of tumor tissue was performed as described previously [18, 19]. Primary antibodies: rabbit-anti-ALDH1A1, 20H2L4, 1:100 (Thermo Fisher Scientific, Waltham, MA, United States), rabbit-anti-CD3, SP7, 1:100 (Novus Biologicals, Centennial, CO, United States), mouse-anti CD20, FMC63, 1:100 (Novus Biologicals), mouse-anti-FOXP3, 2A11G9, 1:100 (Santa Cruz Biotechnology Inc., Dallas, TX, United States). Secondary antibodies: goat-anti-mouse-IgG-Alexa Fluor 555 and donkey-anti-rabbit-IgG-Alexa Fluor 488, 1:400 (both Thermo Fisher Scientific).

Quality detection of human Epstein-Barr virus (EBV) EBER RNA by chromogenic in situ hybridization (CISH) was performed as written in the product protocol (CISH Implementation Kit, Zytovision). ZytoFast EBV probe (PF29) was used, which consists of digoxigenin-labeled oligonucleotides (0.2 ng/µl) targeting mRNA sequences encoding EBER-1 and EBER-2. Various incubation steps were carried out in the programmable ThermoBrite System (Abott Molecular).

### Indirect immunofluorescence staining of spheroids

The cells were fixed in ice cold 4% PFA in a 70% PBS/ 30% H_2_Odd mixture for 25 min. Staining was performed as described previously [6]. Primary antibodies: mouse-anti-CD4, W3/25, 1:100 (Santa Cruz Biotechnology), mouse-anti-CD45-PC7, 1:100 (Beckman Coulter), rabbit-anti-ALDH1A1, 20H2L4, 1:100 (Thermo Fisher Scientific, Waltham, MA, United States), rabbit-anti-CD3, SP7, 1:200 (Novus Biologicals, Centennial, CO, United States), mouse-anti CD20, FMC63, 1:200 (Novus Biologicals), mouse-anti-FOXP3, 2A11G9, 1:100 (Santa Cruz Biotechnology). Secondary antibodies: goat-anti-mouse-IgG-Alexa Fluor 555 and donkey-anti-rabbit-IgG-Alexa Fluor 488, 1:400 (both Thermo Fisher Scientific).

### Xenograft tumor models

All animal experiments are performed following a protocol approved by the Institutional Animal Care and Use Committee of the University of Granada (procedures 11-CEEA-OH-2013), developed in accordance with the European Convention for the Protection of Vertebrate Animals used for Experimental and Other Scientific Purposes (CETS #123) and Spanish law (R.D. 53/2013). This study is conducted using NSG mice (NOD.Cg-Prkdcscid Il2rgtm1Wjl/SzJ, The Jackson Laboratory, Bar Harbor, ME, United States), housed in appropriate sterile filter-capped cages, and fed and given water ad libitum.

After cell recollection, cell viability is assessed using the trypan blue exclusion test. 1–5 × 10^6^ BI-12 tumor cells were resuspended in DMEM (31,053–044, ThermoFisher) and Matrigel matrix (354,234, Corning) at 1:1 ratio. Mice were anesthetized with 3–5% isofluorane in the breathing air. Then, 0.2 mL of this cell suspension was transplanted subcutaneously (s.c.) into the left flank of each mouse. Twice weekly, the animals are monitored for tumor development and tumor sizes are determined using a vernier caliper. Tumor volume is calculated as (width × length^2^)/2. When tumors reach approximately 1 cm^3^, mice are sacrificed by cervical dislocation. Once sacrificed, tumors are digested with collagenase (S1745401, Nordmark Biochemicals) and parts of the tissue fixed in 4% paraformaldehyde (2.529.311.214, PanReac) for 24 h. The fixed samples are dehydrated, cleared and embedded in paraffin following standard protocols [20]. The study is reported in accordance with ARRIVE guidelines.

## Results

### HPV+ HNSCC cells fail to expand in cell culture

**Fig. 1:**
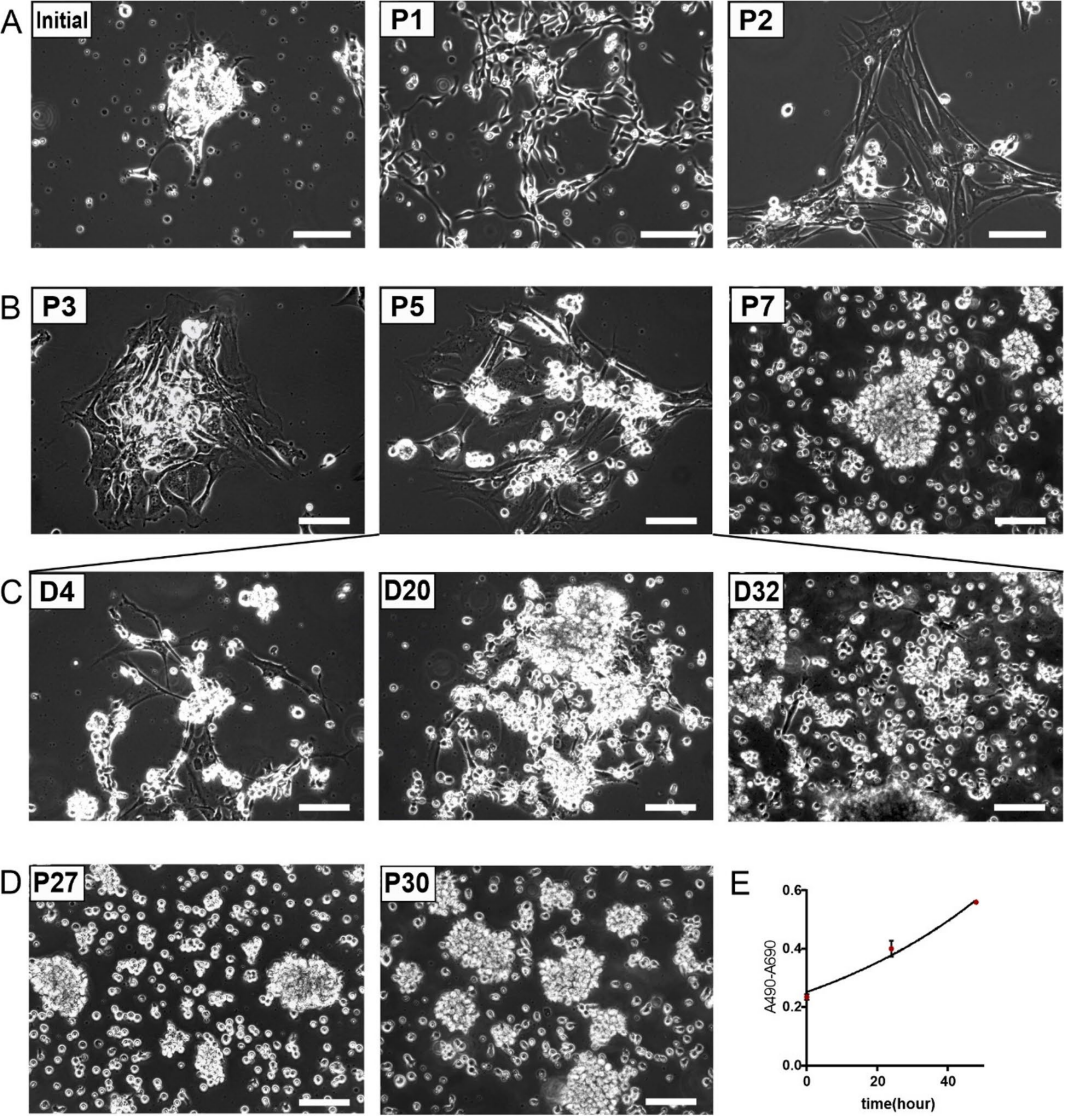
Morphology of BI-12 cells from different cell culture passages. **A** In cell culture passage 2 (P2) attached cells showed mixed epithelial/mesenchymal appearance and single round cells float in suspension or stick to adherent cells. **B** At passage 3 (P3) single cells attach to the extended patches of adherent epithelial cells and fibroblasts. Subsequently, the small cells overgrew the epithelial cells and fibroblasts disappeared. At passage 7 (P7) only small cells were visible forming loosely connected spheroids. **C** A colony of adherent cells in passage 5 imaged over the course of 32 days (D4 = Day 4, D20 = Day 20, and D32 = Day 32). Epithelial cells disappeared while loosely attached round cells expanded as spheroids. **D** Late passages of P27 and P30. **E** Growth curve of the obtained cells depending on MTS assay, the doubling-time was 34.33 ± 4.96 h based on 3 independent triplicate experiments (Suppl. Table S2). P1-P30 = passages 1–30; scale bars = 100 μm

We previously described a system to establish primary HNSCC cell cultures and expand tumor cells under defined conditions using Pneumacult Ex Plus basal medium (PNEU-medium) [6]. Cell culture BI-12 originated from a HPV-positive squamous cell carcinoma of the tonsil which was established in serum-free DMEM-F12 advanced medium and failed to stably grow after culture passage 1 in any medium including PNEU [6]. As epithelial cell proliferation was observed to be much more efficient with subsequent primary cultures which were established directly in PNEU-medium, we attempted again to stably establish culture BI-12 using initially cryopreserved tumor cells. This time, PNEU medium was used from the beginning and cultivation was continued until passage 7 to identify the cell population stabile expanding under these conditions. As observed previously [6], in passages 0 to 1 the majority of cells displayed an epithelial phenotype (Fig. 1A). Similar as described before, in passage 2 three different types of cells were detected, epithelial cells, spindle-shaped cells reminiscent of stromal fibroblasts and single cells with a round morphology, which resided attached to the other two cell types and did not adhere to the ground of the culture dish. In passage 4, the growth of epithelial and fibroblast-like cells was diminished, while the round cells showed strong proliferation and formed suspension spheroids by passage 5. After that, epithelial cells and fibroblasts were lost from the primary culture (Fig. 1B). Interestingly, when we monitored a defined spot of epithelial cells over the course of 32 days at passage 5, we observed that epithelial cells and fibroblasts disappeared gradually, while loosely attached round cells expanded (Fig. 1C, D). This indicates that the epithelial cell population died and was not only simply overgrown by the faster proliferating spheroid cell population. These cells showed immortality due to their ability to proliferate exponentially over 30 passages with a doubling time of 34.33 ± 4.96 h, as determined by MTS assay (Fig. 1E, Suppl. Table S2).

**Fig. 2:**
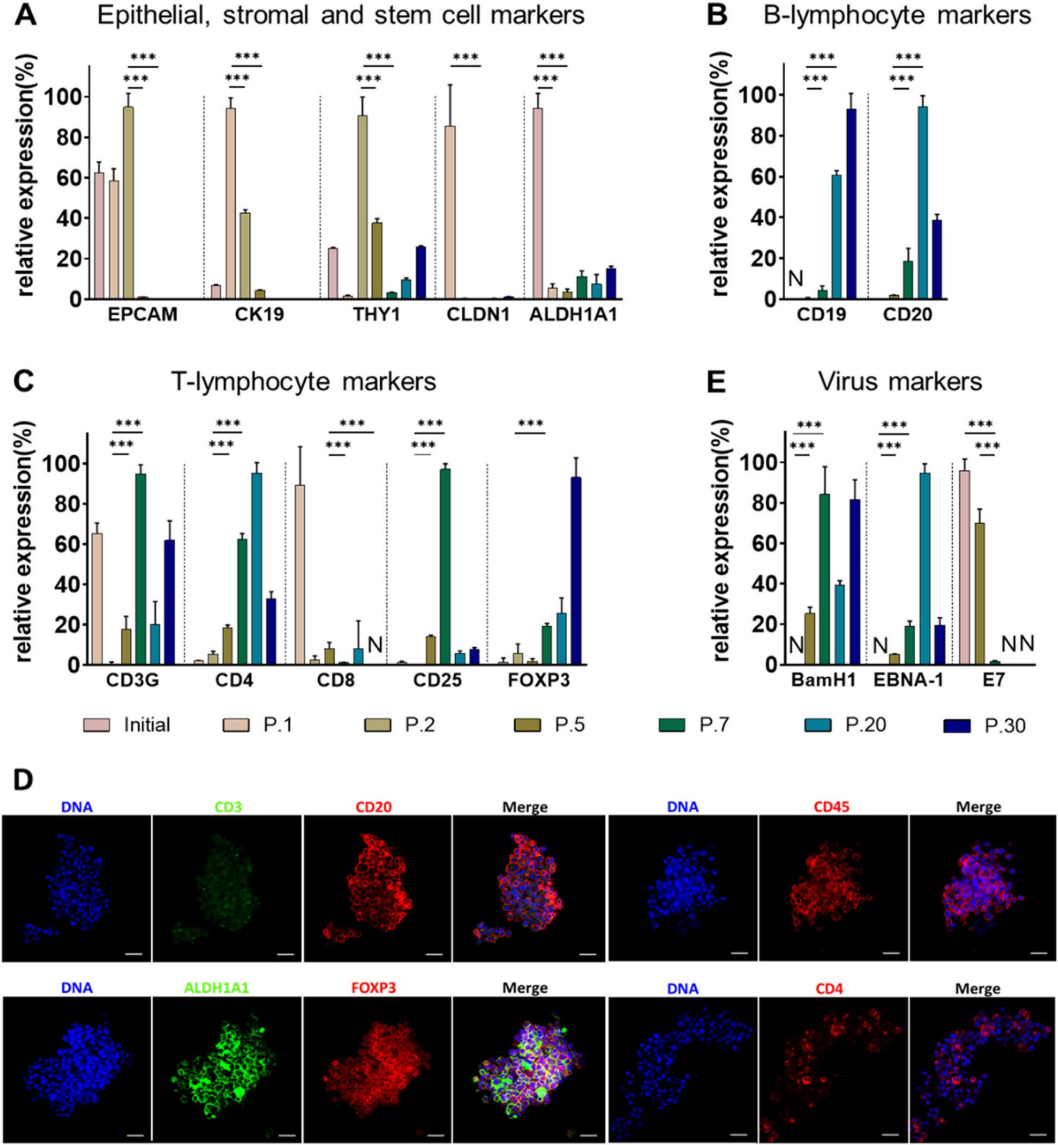
Expression of selected markers determined by RT-qPCR. **A** Epithelial markers EPCAM and CK19 as well as fibroblasts marker THY1 decreased strongly in late passages, similar to cell junction protein CLDN1. Meanwhile, persistent expression of stem cell marker ALDH1A1 indicated the existence of stem-like cells in culture. The increased transcription levels of B cell markers CD19 and CD20 (**B**) and T cell (**C**) markers CD3G, CD4, CD25, and FOXP3 along passages corresponds to accumulation of spheroid-forming cells. The markers indicate three types of T lymphocytes: CD4( +) helper T cells, CD8( +) cytotoxic T cells and CD25( +) FoxP3( +) regulatory T cells (Tregs). **D** Immunofluorescence staining of BI-12 spheroid cells in passage 14. Lymphocyte markers CD45, FOXP3, and CD20 are strongly expressed by most cells, whereas CD4^+^ cells represent a subpopulation. CD3^*dim*^ cells can be observed within the CD20 + population. Staining for stem cell marker ALDH1A1 revealed robust expression. **E** EBV specific DNA fragments *BamHI-W* and *EBNA-1* can be detected and exhibit a tendential increase in association with the markers presented in (**B**) and (**C**). Expression of the HPV oncogene *E7* diminished along the culture passages in correlation with the epithelial markers shown in (**A**); **p < 0.01; ***p < 0.0001; n = 3; *p*-values were determined by Dunnett's multiple comparisons test; P.1-P.30 = passages 1–30; N = not detected; ns = not significant

In our prior study, we describe that EPCAM and CK19 specifically mark epithelial cells in primary HNSCC cell cultures, whereas THY1 can be used as an efficient marker of stromal fibroblast-like cells [6]. In line with the observed disappearance of adherent cells, the expression of EPCAM, CK19, and THY1 simultaneously decreased over the passages until passages 5–7, when these markers were nearly undetectable (Fig. 2A). The same was observed for cell junction marker CLDN1. This indicated that within five cell culture passages epithelial HNSCC cells and stromal fibroblasts were simultaneously lost and overgrown by a third cell type that neither expressed epithelial nor fibroblast markers and exhibited anchorage-independent growth.

### Tumor infiltrating lymphocytes form spheroids under HNSCC cell culture conditions

As undifferentiated cell populations in various malignancies including so called cancer stem cells (CSCs) frequently show the ability to proliferate under serum-free conditions as suspension spheroids, we hypothesized that the here observed spheroids might be comprised of HNSCC cells that have no differentiation marker expression and thus might represent a stem-like subpopulation enriched by serial passaging in PNEU medium. Indeed, the HNSCC stem cell marker ALDH1A1 was expressed [21–23], which we previously reported to be highly expressed in the primary HNSCC spheroid line S-18 established under the same conditions [6].

Furthermore, we tested for these markers in passage 12 and 20, as shown in Suppl. Figure S1, the whole population was still CD45 + , but almost all of them (99%) were characterized as CD19 + CD20 + . Unexpectedly, 12% and 18% of the cells in passage 12 and 20 were also CD3 + , respectively, and the majority of CD3 + subpopulation was CD25 + CD56 + simultaneously. These CD3 + cells appeared as a weakly positive with whole population shifting into the CD3-positive gate. The changes of lymphocyte marker expression on RNA level in different passages were in line with the flow cytometry results, especially the expanding CD19 + CD20 + B cell population at late passages became dominant. Indirect immunofluorescence staining results confirmed the expression of CD20, FOXP3, and ALDH1A1 in these late passage spheroid cells (Fig. 2D). Interestingly, we also observed a weak but systemic staining of CD3 in immunofluorescence which correlated with the weak positive CD3 signal in flow cytometry (Suppl. Figure S1) and the ongoing CD3 expression on RNA level (Fig. 2C).

### Proliferative lymphocytes are EBV-transformed and vulnerable to MDM2 inhibition

Due to the detection of EBV + lymphocytes in previous studies, we tested for the expression of EBV-specific transcripts of the *BamHI-W* and *EBNA-1* region and observed their simultaneous upregulation along passages and sustained expression at late passages (Fig. 2E). In contrast, transcripts of the HPV *E7* fragment decreased at late passages accompanied with the loss of the epithelial component and was not detectable after passage 7. Due to these findings, we concluded that the observed spheroids derived from BI-12 were composed of EBV-infected lymphocytes from the original patient`s tumor tissue.

**Fig. 3:**
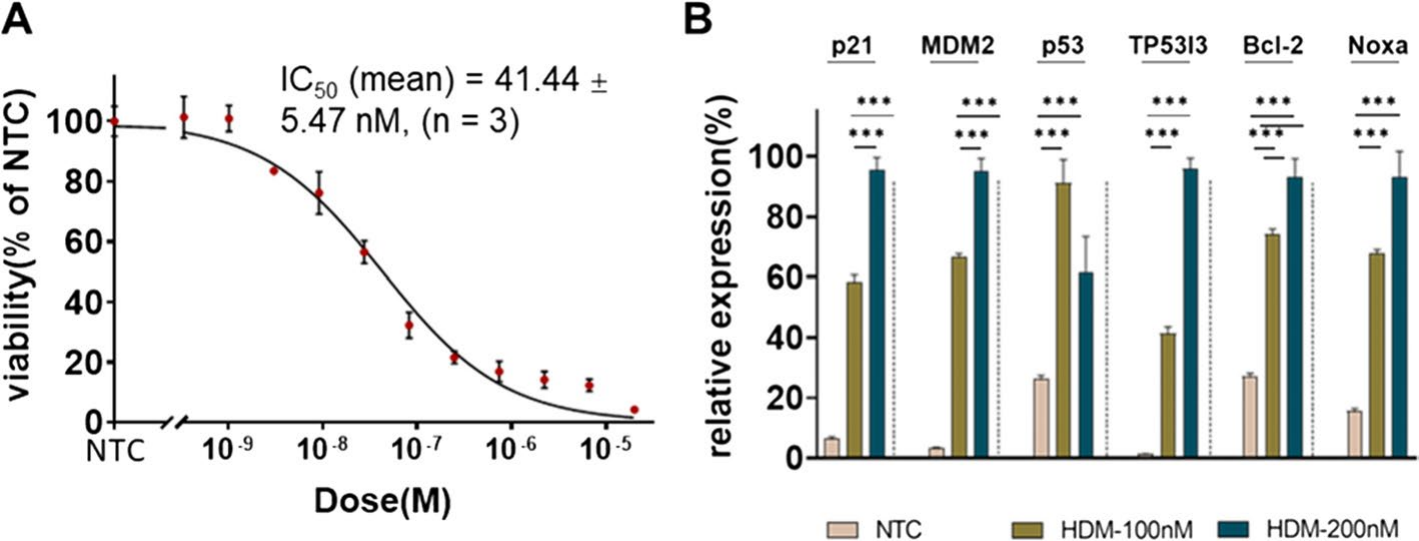
MDM2 inhibitor HDM201 targets BI-12-derived spheroid cells. **A** Representative dose-viability curve of HDM201 treatments, data is shown with *mean* ± *SD* (dot with error bar, n = 3), The half maximal inhibitory concentration (IC50) with 95% confidence interval was calculated in GraphPad Prism by the function of ‘log(inhibitor) vs. normalized response – Variable slope’, detailed data is shown in Suppl. Table S3, based on 3 independently performed experiments, the IC_50_ (mean) was determined as 41.44 ± 5.47 nM. **B** Upregulation of p53 pathway-associated genes *p21, MDM2, TP53I3, Bcl-2* and *Noxa* upon HDM201 treatment for 48 h in a dose-dependent manner; ***p < 0.001; *n* = 3

Since the EBV is an oncovirus and capable of helping infected cells survive by suppressing tumor suppressor p53-associated apoptosis through the p53 pathway [24], and EBV + B-cell-lymphoma is known to be vulnerable to MDM2 inhibition [25], we tested the treatment of these cells using the MDM2-inhibitor HDM201 which suppresses the binding of MDM2 to p53 [26] leading to p53 protein degradation. Interestingly, the maximal effect of the drug was detected at 2-10 μM and its relative IC_50_ value was 41.44 ± 5.47 nM (Fig. 3A). The p53 pathway associated genes *TP53I3*, *Bcl2*, *NOXA*, *CDKN1A*, and *MDM2* were upregulated by HDM201 treatment at transcription level determined by RT-qPCR (Fig. 3B).

### HPV+ BI-12 HNSCC cells initiate tumors in immunodeficient mice

**Fig. 4:**
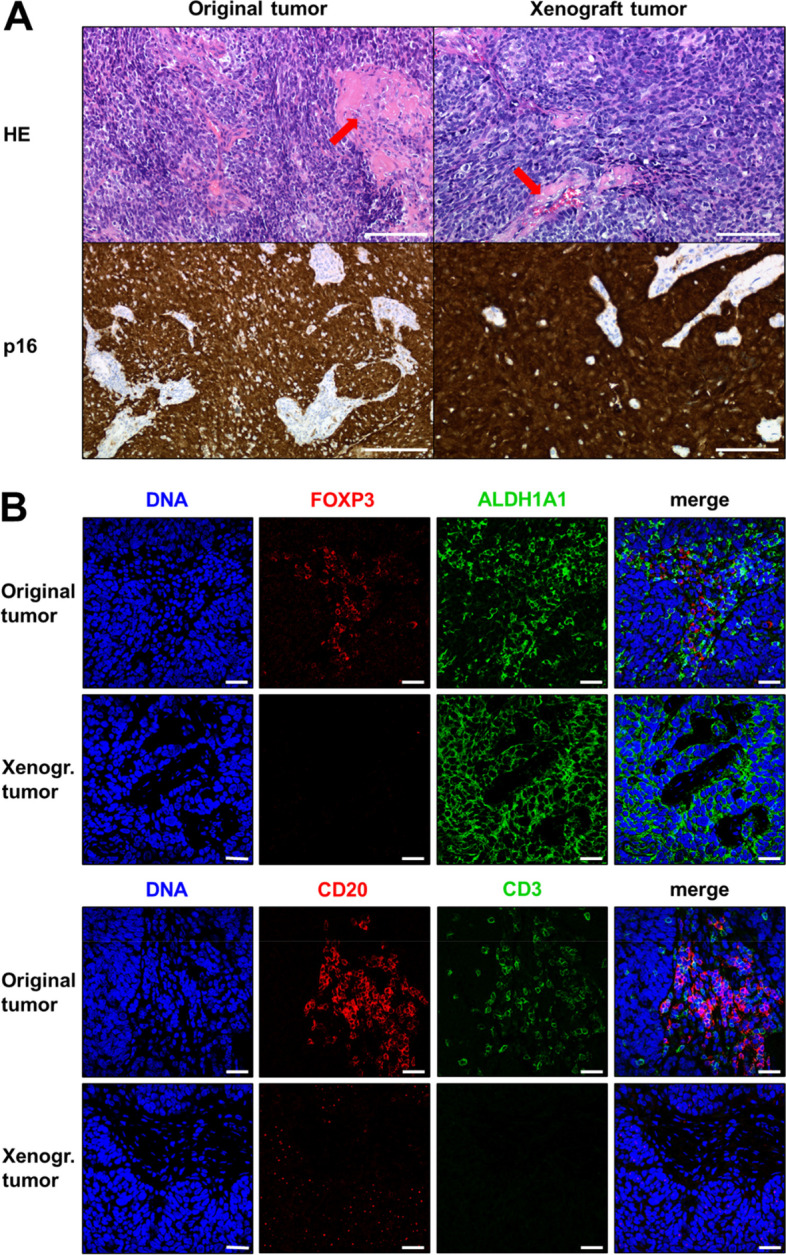
Comparison of the BI-12 xenograft tumor tissue to the original patient`s tumor. **A** HE-staining (top) shows highly similar tissue architecture between original tumor and xenograft tumor. Arrows indicate areas of tumor-associated stroma. Both tumors are highly positive for the HPV surrogate marker p16 (bottom); scale bars = 100 µm. **B** Indirect immunofluorescence staining for HNSCC stem cell marker ALDH1A1 and Treg marker FOXP3 (top) reveals ALDH1A1 + tumor cells in the original tumor and the xenograft tissue. FOXP3 was expressed only in the stromal areas of the patient`s tumor but not in the xenograft. B cell marker CD20 and T cell marker CD3 show B and T lymphocyte infiltration only in the human but not in the xenograft tumor; scale bars = 10 µm

As our model was comprised of HPV + epithelial HNSCC cells unable to expand in cell culture and immortal EBV + lymphocytes proliferating indefinitely in vitro, we were interested to see where these lymphocytes originate from, and which populations were able to form tumors in immunodeficient mice. In a previous study on gastrointestinal tumors, carcinomas, lymphomas, or mixed xenograft tumors were observed in xenograft tumor models in immunodeficient mice [5]. To test for tumorigenicity in both, the epithelial and the lymphocyte compartment, we injected cryopreserved freshly purified BI-12 cells of the original tumor into immunodeficient NSG mice. After 3–5 months, tumor formation was observed and analysis of the tumor tissue revealed that in histology the xenograft tumor well reflected to the original patient`s tumor with strong p16 expression, which represents a surrogate marker for HPV-positivity in clinical practice (Fig. 4A). Single dispersed EBV + cells were found in the original patient’s tumor (Suppl. Figure S2) but not in the xenograft tumor. Original and xenograft tumors displayed patches appearing to be tumor-associated stroma (Fig. 4A, arrows). Immunofluorescence staining of the tissue revealed strong infiltration of the original tumor with CD3 + , CD20 + , and FOXP3 + cells in these patches (Fig. 4B). Some of the FOXP3 + Tregs co-expressed ALDH1A1, as observed in late passage spheroids (Fig. 2). However, despite showing equivalent patches, the xenograft tumor tissue stained totally negative for these human markers. This indicates that the xenograft tumor tissue was not populated by the original human immune cells including the EBV+ spheroid forming subpopulation. Please note that immunodeficient NSG mice by nature are missing mature T, B, and NK cells [27].

## Discussion

### EBV+positive lymphocytes mimic HNSCC spheroids in primary cell culture

The above presented results show that in our in vitro model ALDH1A1-positive EBV-transformed lymphocytes residing in or near the tumor tissue proliferated and phenotypically mimicked HNSCC tumor spheroids. As EBV-transformed lymphocytes are also known to form undifferentiated tumors in immunodeficient mice [5], they can be mistaken functionally as well for epithelial tumor-initiating cells that have undergone epithelial-to-mesenchymal transition (EMT). Obviously, primary HNSCC cell cultures are vulnerable for contaminations not only with stromal fibroblasts as described previously [6], but also can be spoiled by the outgrowth of virally transformed immune cells. Compared to HNSCC cells, these spheroid cells will react differentially towards drug treatment regimen and other biological assays and thus will compromise basic and translational research studies if they remain unidentified, even as a subpopulation.

### EBV-transformed lymphocytes resemble regulatory B cells

The outgrowth of EBV-transformed lymphocytes in our primary HNSCC cell culture as well as the findings on primary tumor tissue indicates that the original cells were in close proximity to the epithelial tumor tissue. TILs are retained in tumor tissue residing adjacent to cancer cells and attracted a lot of attention since they were reported in the 1970s [28, 29]. The cells can be found in various tumor types with a frequency ranging from 16.2% to 100% [30–37] or xenograft tumor tissue [5, 38] (reviewed in Table 1). IL-2 supplemented cell culture conditions are frequently used to establish in vitro models of CD3 + lymphocytes [39–42]. The abundance of TILs within the tumor is diverse from 3 to 74% of all cells [42, 43] with varying sizes of the B or T lymphocyte subpopulation [30, 37]. The stromal compartment can influence TIL composition in the tumor [44]. It was reported that the infiltration of T and B lymphocytes was highly elevated in p16 + HPV-associated HNSCC tissue [30]. Moreover, many studies concluded a positive relationship between presence of TILs and a better outcome for cancer patients [30, 32, 33, 36, 37, 45–48]. Since TILs were naturally retained in cancer tissue, they were believed to recognize tumor-associated antigens leading to the successful strategy to utilize these cells in cancer immunotherapy [39, 42, 49–51]. Altogether, TIL composition is highly context-dependent and various immune cell types can potentially be found in primary cell culture.

**Table 1:** Review of reports on tumor infiltrating lymphocytes

**Cancer type**	**TIL + cases** (% of total cases, number of total cases)	**Features of TIL**	**Prognostic population**	**EBV status**	**Reference**
HNSCC	100% (*n* = 40)	^c^ T11^+^, T4^+^ or T8^+^ Rare B1^+^ or Leu 7^+^	T8^+^ cell	N.T	[32]
	89% of HPV^+^ cases (17/19) 56% to 64% in the rest; 136 cases totally	^c^ CD3^+^, CD8^+^, CD20^+^	CD3^+^ T cell	N.T	[30]
	100% (*n* = 50)	^c^ CD20^+^ B cell	Peritumoral CD20^+^ cell	N.T	[33]
	^c^ 98.8% (*n* = 85)	Not mentioned	Not mentioned	N.T	[34]
	100% (*n* = 5)	^a^ CD3^+^ T cell with CD4^+^ or CD8^+^	Not mentioned	N.T	[39]
	^b^17.8% (8/45 total cases); 50% (8/16 xenografts)	^b^CD20^+^ cell partially with CD3^+^ cells	Not mentioned	8 EBV^+^ cases	[38]
Lung cancer	1/1	unknown	Not mentioned	N.T	[42]
	93.75% (*n* = 32) (Brain metastasis)	^c。^CD3^+^ T cell, CD8^+^, FoxP3^+^	Not mentioned	N.T	[35]
	16.2% (*n* = 155)	^c^ CD3^+^ T cell, CD20^+^ B cell	CD3^+^ cell, CD45^+^ cell, CD20^+^ cell	N.T	[52]
Brest cancer	29.2% (*n* = 788)	^c^ B cell, T cell	B cell	N.T	[36]
	1/1	^a^ CD3^+^ T cell with CD4^+^ or CD8^+^	Not mentioned	N.T	[40]
	1/1	^a^ CD3^+^ T cell with CD4^+^ or CD8^+^	Not mentioned	N.T	[42]
	64.4% (*n* = 90) CD3^+^ 24.4% (*n* = 90) CD20^+^	^c^ B cell, T cell	Not mentioned	N.T	[31]
Cervical and ovarian cancer	1/1	^a^ CD3^+^ T cell with CD4^+^ or CD8^+^	Not mentioned	N.T	[40]
	2/2	^a^ CD3^+^ T cell with CD4^+^ or CD8^+^	Not mentioned	N.T	[42]
	100% (*n* = 18)	^a^ CD3^+^ T cell with CD4^+^ or CD8^+^	Not mentioned	N.T	[39]
Gastrointestinal cancer	1/1	^a^ CD3^+^ T cell with CD4^+^ or CD8^+^	Not mentioned	N.T	[42]
Colorectal cancer	100% (*n* = 67)	^c^ 100% with CD4^+^ CD8^+^ 53.7% with CD20^+^	Not mentioned	N.T	[37]
	1/1	^a^ CD3^+^ T cell with CD8^+^ or CD25^+^	Not mentioned	N.T	[40]
	2/2	^a^ CD3^+^ T cell with CD4^+^ or CD8^+^	Not mentioned	N.T	[42]
	38.3% (*n* = 47)	^b^CD45^+^ cells with CD20^+^	Not mentioned	12 EBV ^+^ cases	[5]
	100% (*n* = 5)	^a^ CD3^+^ T cell with CD4^+^ or CD8^+^	Not mentioned	N.T	[39]
Pancreatic ductal adenocarcinomas	32.4% (*n* = 37)	^b^CD45^+^ cells with CD20^+^	Not mentioned	4 EBV ^+^ cases	[5]
Urinary system cancer	100% (*n* = 9)	^a^ CD3^+^ T cell with CD4^+^ or CD8^+^	Not mentioned	N.T	[40]
	1/1	^a^ CD3^+^ T cell with CD4^+^ or CD8^+^	Not mentioned	N.T	[42]
	1/1	^a,c^ CD20^+^ B cell	Not mentioned	EBV ^+^	[4]
Melanoma	2/2	^a^ CD3^+^ T cell with CD4^+^ or CD8^+^	Not mentioned	N.T	[40]
	100% (*n* = 9)	^a^ CD3^+^ T cell with CD4^+^ or CD8^+^	Not mentioned	N.T	[42]
	100% (*n* = 196)	^a^ T lymphocyte	Not mentioned	N.T	[41]
Sarcoma	1/1	^a^ CD3^+^ T cell with CD8^+^ or CD25^+^	Not mentioned	N.T	[40]
	100% (*n* = 10)	^a^ CD3^+^ T cell with CD4^+^ or CD8^+^	Not mentioned	N.T	[42]

^a^data from TIL cultivation

^b^data form xenograft model

^c^data from immunohistology staining of original tumor tissue; N.T.: not detected

In the here presented case, a mixture of EBV-infected lymphocytes showed delayed in vitro proliferation. Similar findings were reported previously in other tumor entities [4, 5]. Chuang and colleagues prepared RPMI-1640 medium containing 10% heat-inactivated fetal bovine serum to cultivate renal pelvis urothelial carcinoma and, unexpectedly, a population of CD19 + /CD20 + tumor infiltrating B lymphocytes overgrew the adherent cells [4], Interestingly, specific DNA fragments of EBV differed between these cells and peripheral blood mononuclear cells. This indicates that these EBV-positive TILs may represent a distinct population of lymphocytes genetically adapted to the tumor microenvironment. Moreover, the above-mentioned report on gastrointestinal tumors [5] described that in 38.3% (18/47) of patient-derived xenograft tumor models in colorectal carcinoma (CRC) and 10.8% (12/37) in pancreatic ductal adenocarcinoma (PDAC) exhibited CD45 + CD20 + B-lymphocyte proliferation, while EBV-derived RNA was detectable in the CD45 + compartment of 66.7% (12/18) of the CRC cases and in 100% (12/12) of the PDAC tumors. The similar findings on HNSCC were also reported [38], accordingly, 50% (8/16) of xenograft tumors were lymphoma-like tissue with EBV^+^ and CD20^+^, and some of them retains CD3^+^ cells. In our case, cell culture BI-12 exhibited both T- and B-lymphocyte proliferation rather than B-lymphocytes exclusively in early passages, which then was replaced by a stable CD45 + CD19 + CD20 + Foxp3 + main population obtained over 12 passages. This population resembled B regulatory cells (B_regs_) that were previously described [53] and simultaneously expressed ALDH1A1. This unusual marker expression pattern is most likely attributed to the abnormal EBV-transformed nature of these cells. However, to our knowledge this has never been detected before in primary HNSCC cell cultures. The expression of EBV-specific transcripts was detected to increase between passages 5 and 20, correlating with the observed B-cell outgrowth. EBV-positive T-cells were only established from nasal T/NK lymphoma patients using IL-2-supplemented medium [54], whereas all other reports of EBV-positive lymphocytes isolated from tumor tissue mention populations expressing B-cell maker CD20 (Table 1). Thus, EBV-infected spheroid cells in our primary cell cultures appeared to be B- rather than T-lymphocytes.

The pathogen EBV ubiquitously exists in humans and over 90% of adults worldwide that have undergone previous EBV infection [55]. As a confirmed oncovirus, EBV can suppress apoptosis and promote survival and proliferation of infected cells from various histological origins [24]. Infection of lymphoid cells can cause EBV-associated lymphoproliferative disorders, which predominantly impacts B cell and T cell/natural killer (NK) cells [55, 56]. In addition, several reports uncovered the presence of EBV in tumor tissue [57]. As TILs represent a considerable population in human tumor tissue and can contain a potentially large EBV-positive compartment which may be sensitive to MDM2 inhibition, more attention should be paid to the impact of tumor-associated EBV + lymphocytes on cancer biology. However, the poor test rate (18.7%, 3 in 16 reports, reviewed in Table 1) for EBV in studies on TIL populations highlights the importance of investigating EBV infection in primary tumor tissue for cancer research and clinical applications.

### BI-12 HPV+ HNSCC cells induce tumors in immunodeficient mice

The epithelial BI-12 tumor cells originally isolated from the patient-derived tissue did not expand beyond initial passages and were overgrown by lymphocytes. This questions the malignant origin of these epithelial cells, as it is known that non-malignant epithelial cells can outcompete HNSCC cells in culture establishment [58]. However, expression of the HPV-E7 oncogene points towards a malignant origin of these cells which were isolated from an HPV-positive HNSCC. Moreover, transplantation of freshly purified BI-12 tumor cells into immunodeficient mice resulted in xenograft tumor formation with HNSCC histology and strong p16 expression that appeared identical to the original patient`s tumor and contained no detectable human lymphocyte population. Thus, the EBV + lymphocytes in the patient’s tumor grew up in cell culture but did not engraft into the in vivo model. EBV + lymphocytes from primary tumor tissue are known to frequently engraft into xenograft tumor models, but not in every case [5, 38]. The biological factors that influence the engraftment of these cells in immunodeficient mice are poorly understood and further research would be necessary to define them. Genetic/epigenetic variants might be involved in our case, so that the overall fitness of the EBV-transformed lymphocytes was not sufficient to engraft. In contrast, the epithelial HPV + HNSCC cells failed to expand in vitro but formed tumors in NSG mice. This suggests how sensitive primary cells can react towards alterations in their environment, even though they were transformed by an oncovirus.

EBV+ lymphoma-like tumor formation is a known problem in the establishment of xenograft tumor models from HPV + HNSCC tissue [38]. Even though it is not excluded that EBV + lymphoproliferation is observed in later xenograft tumor generations generated from our model, at this stage it appears to closely reflect the characteristics of the patient`s tumor and thus represents a suitable model to test targeting strategies based on immunotherapy approaches or molecular targeted drugs in future studies. The faithfully retained p16 expression indicates ongoing HPV activity, as p16 expression is not only used as surrogate marker for HPV-positivity in clinics but was demonstrated in previous study to correlate well with HPV-RNA expression [59].

## Conclusions

Altogether, our observation exemplifies a serious flaw in primary HNSCC cell culture, which can significantly distort research projects if left undetected. Additionally, we also report a primary cell culture of EBV-driven B-lymphocytes and an HPV + HNSCC xenograft tumor model that might be valuable for future research projects. We also highlight the importance to test for EBV whenever TILs are used as research model or in clinics. Primary tumor cell culture is challenging as benign cell populations can resemble tumor cells in morphology and marker expression. For instance, fibroblasts can be confused with scattered epithelial-to-mesenchymal transition-like tumor cells, benign epithelial cells from normal tissue attached to primary tumor samples can mimic carcinoma cells, and—as demonstrated here—lymphocytes can appear as tumor spheroids. The recent progress computational cancer biology, molecular targeting using specific inhibitors, and genome editing technology awoke hope for new highly effective and tumor-specific anti-cancer treatments in near future. However, in order to translate powerful research tools into therapies, faithful and unbiased preclinical models are needed.

## Supplementary Information

**Additional file 1:** **Supplementary Figure S1.** Expression of lymphocyte markers in different passages. **Supplementary Figure S2.** In situ hybridization of EBV. **Supplementary Table S1.** RT-qPCR primers purchased from Sigma-Aldrich. **Supplementary Table S2.** Data of BI-12 growth curve. **Supplementary Table S3.** Data of HDM201 treatment in BI-12 cells.

## Data Availability

All data generated or analyzed during this study are included in this published article and its supplementary information files.

## References

[1] Bray F, Ferlay J, Soerjomataram I, Siegel RL, Torre LA, Jemal A (2018). Global cancer statistics 2018: GLOBOCAN estimates of incidence and mortality worldwide for 36 cancers in 185 countries. CA: a Cancer J Clin.

[2] Pastor DM, Poritz LS, Olson TL, Kline CL, Harris LR, Koltun WA (2010). Primary cell lines: false representation or model system? a comparison of four human colorectal tumors and their coordinately established cell lines. Int J Clini Experiment Med.

[3] Gottschling S, Jauch A, Kuner R, Herpel E, Mueller-Decker K, Schnabel PA (2012). Establishment and comparative characterization of novel squamous cell non-small cell lung cancer cell lines and their corresponding tumor tissue. Lung Cancer.

[4] Chuang CK, Chuang KL, Hsieh CH, Shen YC, Liao SK (2010). Epstein-Barr virus-infected cell line TCC36B derived from B lymphocytes infiltrating renal pelvis urothelial carcinoma. Anticancer Res.

[5] Dieter SM, Giessler KM, Kriegsmann M, Dubash TD, Mohrmann L, Schulz ER (2017). Patient-derived xenografts of gastrointestinal cancers are susceptible to rapid and delayed B-lymphoproliferation. Int J Cancer.

[6] Oppel F, Shao S, Schurmann M, Goon P, Albers AE, Sudhoff H. An Effective Primary Head and Neck Squamous Cell Carcinoma In Vitro Model. Cells. 2019 Jun 7;8(6). PubMed PMID: 31181618. Pubmed Central PMCID: 6628367. 10.3390/cells8060555 PMC6628367 31181618

[7] Kamangar F, Dores GM, Anderson WF (2006). Patterns of cancer incidence, mortality, and prevalence across five continents: defining priorities to reduce cancer disparities in different geographic regions of the world. Journal of clinical oncology : official journal of the American Society of Clinical Oncology.

[8] Ferlay J, Soerjomataram I, Dikshit R, Eser S, Mathers C, Rebelo M (2015). Cancer incidence and mortality worldwide: sources, methods and major patterns in GLOBOCAN 2012. Int J Cancer.

[9] Leemans CR, Snijders PJF, Brakenhoff RH (2018). The molecular landscape of head and neck cancer. Nat Rev Cancer.

[10] Galot R, Le Tourneau C, Guigay J, Licitra L, Tinhofer I, Kong A (2018). Personalized biomarker-based treatment strategy for patients with squamous cell carcinoma of the head and neck: EORTC position and approach. Anna Oncol : Official J European Soc Med Oncol.

[11] Hanahan D (2022). Hallmarks of Cancer: New Dimensions. Cancer Discov.

[12] Worsham MJ, Wolman SR, Carey TE, Zarbo RJ, Benninger MS, Van Dyke DL (1999). Chromosomal aberrations identified in culture of squamous carcinomas are confirmed by fluorescence in situ hybridisation. Mol Pathol : MP.

[13] Ragin CC, Reshmi SC, Gollin SM (2004). Mapping and analysis of HPV16 integration sites in a head and neck cancer cell line. Int J Cancer.

[14] Chiou SH, Yu CC, Huang CY, Lin SC, Liu CJ, Tsai TH (2008). Positive correlations of Oct-4 and Nanog in oral cancer stem-like cells and high-grade oral squamous cell carcinoma. Clin Can Res : an official journal of the American Association for Cancer Research.

[15] Kadletz L, Heiduschka G, Domayer J, Schmid R, Enzenhofer E, Thurnher D (2015). Evaluation of spheroid head and neck squamous cell carcinoma cell models in comparison to monolayer cultures. Oncol Lett.

[16] Ishiguro T, Ohata H, Sato A, Yamawaki K, Enomoto T, Okamoto K (2017). Tumor-derived spheroids: Relevance to cancer stem cells and clinical applications. Cancer Sci.

[17] Riss TL, Moravec RA, Niles AL, Duellman S, Benink HA, Worzella TJ, et al. Cell Viability Assays. In: Markossian S, Grossman A, Brimacombe K, Arkin M, Auld D, Austin CP, et al., editors. Assay Guidance Manual. Bethesda: 2004. (PubMed PMID: 23805433).

[18] Ball CR, Oppel F, Ehrenberg KR, Dubash TD, Dieter SM, Hoffmann CM, Abel U, Herbst F, Koch M, Werner J, Bergmann F, Ishaque N, Schmidt M, von Kalle C, Scholl C, Fröhling S, Brors B, Weichert W, Weitz J, Glimm H. Succession of transiently active tumor-initiating cell clones in human pancreatic cancer xenografts. EMBO Mol Med. 2017;9(7):918-932. (PubMed PMID: 28526679. Pubmed Central PMCID: PMC5494525). 10.15252/emmm.201607354 PMC5494525 28526679

[19] Oppel F, Tao T, Shi H, Ross KN, Zimmerman MW, He S, Tong G, Aster JC, Look AT. Loss of atrx cooperates with p53-deficiency to promote the development of sarcomas and other malignancies. PLoS Genet. 2019;15(4):e1008039. (PubMed PMID: 30970016. Pubmed Central PMCID: PMC6476535). 10.1371/journal.pgen.1008039 PMC6476535 30970016

[20] Shen YQ, Guerra-Librero A, Fernandez-Gil BI, Florido J, Garcia-Lopez S, Martinez-Ruiz L, et al. Combination of melatonin and rapamycin for head and neck cancer therapy: Suppression of AKT/mTOR pathway activation, and activation of mitophagy and apoptosis via mitochondrial function regulation. J Pineal Res. 2018;64(3). (PubMed PMID: 29247557). 10.1111/jpi.12461 29247557

[21] Tomita H, Tanaka K, Tanaka T, Hara A (2016). Aldehyde dehydrogenase 1A1 in stem cells and cancer. Oncotarget.

[22] Chen YC, Chen YW, Hsu HS, Tseng LM, Huang PI, Lu KH (2009). Aldehyde dehydrogenase 1 is a putative marker for cancer stem cells in head and neck squamous cancer. Biochem Biophys Res Commun.

[23] Clay MR, Tabor M, Owen JH, Carey TE, Bradford CR, Wolf GT (2010). Single-marker identification of head and neck squamous cell carcinoma cancer stem cells with aldehyde dehydrogenase. Head & neck.

[24] Tornesello ML, Annunziata C, Tornesello AL, Buonaguro L, Buonaguro FM. Human Oncoviruses and p53 Tumor Suppressor Pathway Deregulation at the Origin of Human Cancers. Cancers (Basel). 2018;10(7):213. (PubMed PMID: 29932446. Pubmed Central PMCID: 6071257). 10.3390/cancers10070213 PMC6071257 29932446

[25] Zhang X, Zhang R, Ren C, Xu Y, Wu S, Meng C, et al. Epstein Barr virus-positive B-cell lymphoma is highly vulnerable to MDM2 inhibitors in vivo. Blood Adv. 2022;6(3):891-901. (PubMed PMID: 34861697). 10.1182/bloodadvances.2021006156 PMC8945299 34861697

[26] Thérèse Stachyra-Valat, Frédéric Baysang, Anne-Cécile D’Alessandro, Erdmann Dirk, Pascal Furet, Vito Guagnano, Joerg Kallen, Lukas Leder, Robert Mah, Keiichi Masuya, Stefan Stutz, Andrea Vaupel, Francesco Hofmann, Patrick Chène, Sébastien Jeay, Philipp Holzer. NVP-HDM201: Biochemical and biophysical profile of a novel highly potent and selective PPI inhibitor of p53-Mdm2. [abstract]. In: Proceedings of the 107th Annual Meeting of the American Association for Cancer Research; 2016 Apr 16-20; New Orleans, LA, Philadelphia: AACR; Cancer Res. 2016;76(14 Suppl):Abstract nr 1239.

[27] Shultz LD, Lyons BL, Burzenski LM, Gott B, Chen X, Chaleff S (2005). Human lymphoid and myeloid cell development in NOD/LtSz-scid IL2R gamma null mice engrafted with mobilized human hemopoietic stem cells. J Immunol.

[28] Sjogren HO, Jonsson N (1970). Cellular immunity to Rous sarcoma in tumor-bearing chickens. Can Res.

[29] Wainberg MA, Markson Y, Weiss DW, Doljanski F (1974). Cellular immunity against Rous sarcomas of chickens Preferential reactivity against autochthonous target cells as determined by lymphocyte adherence and cytotoxicity tests in vitro. Proc Natl Acad Sci U S A.

[30] Schneider K, Marbaix E, Bouzin C, Hamoir M, Mahy P, Bol V (2018). Immune cell infiltration in head and neck squamous cell carcinoma and patient outcome: a retrospective study. Acta Oncol.

[31] Marsigliante S, Biscozzo L, Marra A, Nicolardi G, Leo G, Lobreglio GB (1999). Computerised counting of tumour infiltrating lymphocytes in 90 breast cancer specimens. Cancer Lett.

[32] Wolf GT, Hudson JL, Peterson KA, Miller HL, McClatchey KD (1986). Lymphocyte subpopulations infiltrating squamous carcinomas of the head and neck: correlations with extent of tumor and prognosis. Otolaryngol Head Neck Surg : official J American Acad Otolaryngol-Head Neck Surg.

[33] Taghavi N, Mohsenifar Z, Baghban AA, Arjomandkhah A (2018). CD20+ Tumor Infiltrating B Lymphocyte in Oral Squamous Cell Carcinoma: Correlation with Clinicopathologic Characteristics and Heat Shock Protein 70 Expression. Patholog Res Int.

[34] Zhu N, Ding L, Fu Y, Yang Y, Chen S, Chen W, et al. Tumor-infiltrating lymphocyte-derived MLL2 independently predicts disease-free survival for patients with early-stage oral squamous cell carcinoma. J Oral Pathol Med. 2020;49(2):126–36. (PubMed PMID: 31660637). 10.1111/jop.12969 31660637

[35] Berghoff AS, Ricken G, Wilhelm D, Rajky O, Widhalm G, Dieckmann K (2016). Tumor infiltrating lymphocytes and PD-L1 expression in brain metastases of small cell lung cancer (SCLC). J Neurooncol.

[36] Schmidt M, Bohm D, von Torne C, Steiner E, Puhl A, Pilch H (2008). The humoral immune system has a key prognostic impact in node-negative breast cancer. Can Res.

[37] Liao Y, Ou J, Deng J, Geng P, Zeng R, Tian Y (2013). Clinical implications of the tumor-infiltrating lymphocyte subsets in colorectal cancer. Med Oncol.

[38] Facompre ND, Sahu V, Montone KT, Harmeyer KM, Nakagawa H, Rustgi AK (2017). Barriers to generating PDX models of HPV-related head and neck cancer. The Laryngoscope.

[39] Stevanovic S, Helman SR, Wunderlich JR, Langhan MM, Doran SL, Kwong MLM (2019). A Phase II Study of Tumor-infiltrating Lymphocyte Therapy for Human Papillomavirus-associated Epithelial Cancers. Clin Can Res : an official J American Assoc Cancer Res.

[40] Schoof DD, Jung SE, Eberlein TJ (1989). Human tumor-infiltrating lymphocyte (TIL) cytotoxicity facilitated by anti-T-cell receptor antibody. Int J Cancer.

[41] Zippel D, Friedman-Eldar O, Rayman S, Hazzan D, Nissan A, Schtrechman G (2019). Tissue Harvesting for Adoptive Tumor Infiltrating Lymphocyte Therapy in Metastatic Melanoma. Anticancer Res.

[42] Topalian SL, Muul LM, Solomon D, Rosenberg SA (1987). Expansion of human tumor infiltrating lymphocytes for use in immunotherapy trials. J Immunol Methods.

[43] Balch CM, Riley LB, Bae YJ, Salmeron MA, Platsoucas CD, von Eschenbach A (1990). Patterns of human tumor-infiltrating lymphocytes in 120 human cancers. Arch Surg.

[44] Koeck S, Kern J, Zwierzina M, Gamerith G, Lorenz E, Sopper S (2017). The influence of stromal cells and tumor-microenvironment-derived cytokines and chemokines on CD3(+)CD8(+) tumor infiltrating lymphocyte subpopulations. Oncoimmunology.

[45] Mella M, Kauppila JH, Karihtala P, Lehenkari P, Jukkola-Vuorinen A, Soini Y (2015). Tumor infiltrating CD8(+) T lymphocyte count is independent of tumor TLR9 status in treatment naive triple negative breast cancer and renal cell carcinoma. Oncoimmunology.

[46] Shibutani M, Maeda K, Nagahara H, Fukuoka T, Nakao S, Matsutani S (2017). The Prognostic Significance of the Tumor-infiltrating Programmed Cell Death-1(+) to CD8(+) Lymphocyte Ratio in Patients with Colorectal Cancer. Anticancer Res.

[47] Kadota K, Nitadori JI, Ujiie H, Buitrago DH, Woo KM, Sima CS (2015). Prognostic Impact of Immune Microenvironment in Lung Squamous Cell Carcinoma: Tumor-Infiltrating CD10+ Neutrophil/CD20+ Lymphocyte Ratio as an Independent Prognostic Factor. J Thorac Oncol : Official Pub Int Assoc Study Lung Cancer.

[48] VaziriFard E, Ali Y, Wang XI, Saluja K, HC M, Wang L (2019). Tumor-Infiltrating Lymphocyte Volume Is a Better Predictor of Disease-Free Survival Than Stromal Tumor-Infiltrating Lymphocytes in Invasive Breast Carcinoma. American J Clin Pathol.

[49] Robbins PF (2017). Tumor-Infiltrating Lymphocyte Therapy and Neoantigens. Cancer J.

[50] Garber K (2019). Pursuit of tumor-infiltrating lymphocyte immunotherapy speeds up. Nat Biotechnol.

[51] Nelson BH (2010). CD20+ B cells: the other tumor-infiltrating lymphocytes. J Immunol.

[52] Zhao X, Kallakury B, Chahine JJ, Hartmann D, Zhang Y, Chen Y (2019). Surgical Resection of SCLC: Prognostic Factors and the Tumor Microenvironment. J Thorac Oncol : official publication of the International Association for the Study of Lung Cancer.

[53] Vadasz Z, Toubi E (2017). FoxP3 Expression in Macrophages, Cancer, and B Cells-Is It Real?. Clin Rev Allergy Immunol.

[54] Tsuchiyama J, Yoshino T, Mori M, Kondoh E, Oka T, Akagi T (1998). Characterization of a novel human natural killer-cell line (NK-YS) established from natural killer cell lymphoma/leukemia associated with Epstein-Barr virus infection. Blood.

[55] Rezk SA, Zhao X, Weiss LM (2018). Epstein-Barr virus (EBV)-associated lymphoid proliferations, a 2018 update. Hum Pathol.

[56] Kim HJ, Ko YH, Kim JE, Lee SS, Lee H, Park G (2017). Epstein-Barr Virus-Associated Lymphoproliferative Disorders: Review and Update on 2016 WHO Classification. J Pathol Transl Med.

[57] Jha HC, Pei Y, Robertson ES (2016). Epstein-Barr Virus: Diseases Linked to Infection and Transformation. Front Microbiol.

[58] Yu F, Lu Y, Tao L, Jiang YY, Lin DC, Wang L (2017). Non-malignant epithelial cells preferentially proliferate from nasopharyngeal carcinoma biopsy cultured under conditionally reprogrammed conditions. Sci Rep.

[59] Lilja-Fischer JK, Ulhoi BP, Alsner J, Stougaard M, Thomsen MS, Busk M (2019). Characterization and radiosensitivity of HPV-related oropharyngeal squamous cell carcinoma patient-derived xenografts. Acta Oncol.

