# Supplementary material for: Primary head and neck cancer cell cultures are susceptible to proliferation of Epstein-Barr virus infected lymphocytes
Source: BMC Cancer. 2023 Jan 13;23:47. doi: 10.1186/s12885-022-10481-y (PMC9840248; doi:10.1186/s12885-022-10481-y)
Supplement: Supplementary file 1 — Additional file 1: Supplementary Figure S1. Expression of lymphocyte markers in different passages. Supplementary Figure S2. In situ hybridization of EBV. Supplementary Table S1. RT-qPCR primers purchased from Sigma-Aldrich. Supplementary Table S2. Data of BI-12 growth curve. Supplementary Table S3. Data of HDM201 treatment in BI-12 cells. [file 12885_2022_10481_MOESM1_ESM.pdf]

Supplementary material

Supplementary Figure S1: Expression of lymphocyte markers in different passages

Supplementary Figure S2: In situ hybridization of EBV

Supplementary Table S1: RT-qPCR primers purchased from Sigma-Aldrich

Supplementary Table S2: Data of BI-12 growth curve

Supplementary Table S3: Data of HDM201 treatment in BI-12 cells

A

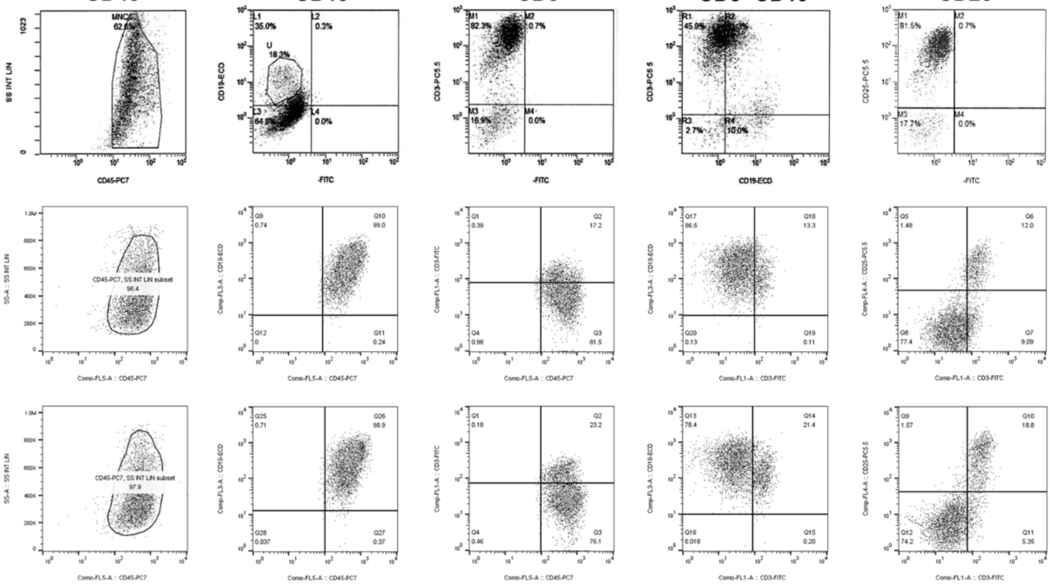

B

Markers	P5	P12	P20
$CD45^+$	63.9%	96.4%	97.9%
$CD45^+CD3^+$	81.5%	17.2%	23.0%
$CD45^+CD3^+CD25^+$	——	12.0%	18.8%
$CD45^+CD19^+$	18.3%	99.0%	99.0%
$CD45^+CD19^+CD20^+$		99.1%	99.3%
$CD45^+CD19^+CD3^+$	——	13.3%	21.0%

Supplementary Figure S1: Expression of lymphocyte markers in different passages. (A) Flow cytometry graphs display the markers CD45, CD3, CD19, and CD25 in single staining and in combination with CD3 and CD19. The main population is $CD45^+$ indicating peripheral blood cells all 3 passages. At passage 5, distinct two subpopulations are detected, $CD3^+$ T cells and $CD19^+$ B cells. The $CD19^-$ population was comprised of $CD3^+CD25^+$ cells reminiscent of regulatory T cells (Tregs). The ratio between T and B lymphocytes was determined to be approximately 4:1. Unexpectedly, at passage 12 and 30 the whole $CD45^+$ population stained $CD19^+$ of which 13.3% and 21% of the cells, respectively, were simultaneously $CD3^+$. The size of these populations is summarized in table **(B)**.

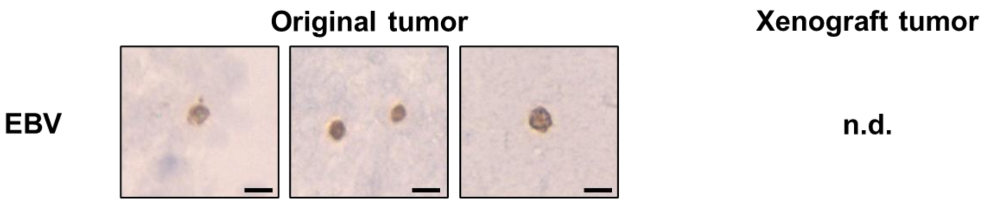

Supplementary Figure S2: In situ hybridization of EBV. Single dispersed EBV+ cells were found in the original patient`s tumor; three representative spots are shown; scale bars = 10µm. These cells were not detected (n.d.) in xenograft tumor tissue in immunodeficient mice.

Supplementary Table S1: RT-qPCR primers purchased from Sigma-Aldrich.

**Primer**	**5' -> 3' sequence**
ALDH1A1 forward	TTGTTCCTGGTTATGGGCCT
ALDH1A1 reverse	GCTGGCAATGCAGACATTCTTA
BamHI-W forward	CCCAACACTCCACCACACC
BamHI-W reverse	TCTTAGGAGCTGTCCGAGGG
Bcl-2 forward	AGAGCGTCAACCGGGAGATGT
Bcl-2 reverse	TGCCGGTTCAGGTACTCAGTCA
CD19 forward	GACAGTCAATGTGGAGGGCA
CD19 reverse	ACACATACAGCTTGGGGCTC
CD20 forward	CGTGCTCCAGACCCAAATCT
CD20 reverse	TCCTGATCTTGGGGAGGTTCT
CD25 forward	TATCATTTCGTGGTGGGGCA
CD25 reverse	GGGTCATTTTGCAGACGCTC
CD3G forward	TCTCTGGAGGTATTCTAGGCAT
CD3G reverse	TAAAAGATAGGGGAAGGGAGGA
CD4 forward	AAGAAAGTGGTGCTGGGCAA
CD4 reverse	TCAGCGCGATCATTCAGCTT

CD8 forward	GCTGGACTTCGCCTGTGATA
CD8 reverse	GGGCTTGTCTCCCGATTTGA
p21 forward	AGTCAGTTCCTTGTGGAGCC
p21 reverse	GCATGGGTTCTGACGGACAT
CK-19 forward	GAATCGCAGCTTCTGAGACCA
CK-19 reverse	CTGGCGATAGCTGTAGGAAGT
CLDN1 forward	AGCTGTTGGGCTTCATTCTC
CLDN1 reverse	CTGGGCGGTCACGATGTT
EBNA-1 forward	TCATCATCATCCGGGTCTCC
EBNA-1 reverse	CCTACAGGGTGGAAAAATGGC
EPCAM forward	TAAGGCCAAGCAGTGCAACG
EPCAM reverse	TTGTCTGTTCTTCTGACCCCAG
GAPDH forward	CTGCACCACCAACTGCTTAG
GAPDH reverse	GTCTTCTGGGTGGCAGTGAT
HPV E7 forward	ATATATGTTACATTTGCAACCAGAGACAAC
HPV E7 reverse	GTCTACGTGTGTGCTTTGTACGCAC
MDM2 forward	GGTGCTGTAACCACCTCACA
MDM2 reverse	TTTTGTGCACCAACAGACTTT
NOXA forward	TTCTTCGGTCACTACACAACG
NOXA reverse	TAACGCCCAACAGGAACACA
THY-1 forward	CAGCAGTTCACCCATCCAGT
THY-1 reverse	TGGTGAAGTTGGTTCGGGAG
p53 forward	CATGGCCATCTACAAGCAGTCACA
p53 reverse	TTGAGTTCCAAGGCCTCATTCAGC
TP5313 forward	ACATCAATGGGCCCCTGTTT
TP5313 reverse	AGGCAGAATTTGCTCCGTGA

Supplementary Table S2: Data of BI-12 growth curve

Repeats	1st	2nd	3rd
doubling time(hour)	40.86	28.86	33.25
95% confidence interval	35.1 to 48.46	24.68 to 34.18	27.06 to 42.05
R^2	0.9735	0.9765	0.9555

Data of growth curve, viability was determined by MTS assay (n = 3), growth medium was used as blank control to subtract background.

Supplementary Table S3: Data of HDM201 treatment in BI-12 cells

Repeats	1st	2nd	3rd
SC	6.67μM	20μM	33.3μM
DF	3	3	3
IC50(nM)	44.79	35.12	44.40
95% confidence interval	38.98 to 51.41	29.54 to 41.81	38.80 to 50.77
Hill Slope	-0.8218	-0.7654	-0.7736
R^2	0.9854	0.9788	0.9863

SC: start concentration; DF: dilution factor

Data of dose-viability of HDM201 treatments, viability was determined by MTS assay (n = 4), growth medium was used as blank control to subtract background.
